# Burden and Impact of Pneumococcal Pneumonia on Outcomes in Patients With Cirrhosis: A Nationwide Analysis

**DOI:** 10.14740/gr2139

**Published:** 2026-04-27

**Authors:** Mariam Alamgir, Nishma Dhand, Isha Kohli, Carol Singh, Aalam Sohal, Nilofar Najafian

**Affiliations:** a School of Medicine, St. George’s University, Grenada, West Indies; b Department of Internal Medicine, Dayanand Medical College and Hospital, Ludhiana, India; c Graduate Program in Public Health, Icahn School of Medicine at Mount Sinai, New York, NY, USA; d Division of Gastroenterology and Hepatology, Creighton University School of Medicine, Phoenix, AZ, USA

**Keywords:** Cirrhosis, Pneumococcal pneumonia, *Streptococcus pneumoniae*, National Inpatient Sample, In-hospital mortality, Healthcare burden

## Abstract

**Background:**

Patients with cirrhosis have impaired immunity, predisposing them to severe infections. *Streptococcus pneumoniae*, a leading cause of community-acquired pneumonia, may worsen outcomes in this vulnerable patient population. This study aims to evaluate the burden and impact of pneumococcal pneumonia among patients with cirrhosis.

**Methods:**

The National Inpatient Sample database (2016–2022) was used to identify adult hospitalizations with cirrhosis. Patients were stratified by the presence or absence of pneumococcal pneumonia. Data were obtained on demographics, liver disease etiology and decompensations, comorbidities, and clinical outcomes. A multivariate logistic/linear regression analysis was used to assess the impact of pneumococcal pneumonia on clinical outcomes.

**Results:**

Among 4,716,863 patients with cirrhosis, 90,680 (1.92%) developed pneumococcal pneumonia. Patients with pneumococcal pneumonia had higher odds of in-hospital mortality (adjusted odds ratio (aOR): 2.95, 95% confidence interval (CI): 2.84–3.09), acute kidney injury (aOR: 1.85, 95% CI: 1.79–1.91), shock (aOR: 4.75, 95% CI: 4.58–4.93), intensive care unit admissions (aOR: 7.55, 95% CI: 7.28–7.83), non-home discharges (aOR: 2.29, 95% CI: 2.21–2.38), longer length of stay (adjusted coefficient: 6.61 days, 95% CI: 6.38–6.83), and higher hospitalization charges (adjusted coefficient: $112,230.5, 95% CI: $106,802.3–$117,658.7) (all P < 0.001).

**Conclusion:**

We noted an increased in-hospital mortality and higher resource utilization among patients with pneumococcal pneumonia. These findings underscore the importance of targeted preventive strategies, including pneumococcal vaccination and early infection recognition, to reduce morbidity and healthcare burden in this vulnerable population.

## Introduction

Liver cirrhosis is a leading cause of global mortality, often necessitating hospitalization for disease-related complications [1–3]. It remains a significant health burden due to immune dysfunction, which considerably increases susceptibility to infections [4]. A meta-analysis has demonstrated that infections in patients with cirrhosis are associated with a roughly four-fold increase in mortality [5]. As such, patients with cirrhosis with bacterial infections are more prone to decompensation, becoming a major contributor to mortality [6–9]. The immune system impairment caused by cirrhosis renders the host vulnerable to bacterial infections and dysregulated immune cell activation [4]. A previous study by Hung et al found that pneumonia was associated with higher mortality among cirrhotic patients with ascites [10]. Cirrhosis-associated defects in innate pulmonary immunity contribute to increased mortality from pneumonia in cirrhotic patients [5, 11]. Furthermore, previous studies have highlighted that pneumonia has been an important cause of septic shock and acute renal failure in patients with cirrhosis [12, 13]. Another study emphasized that among patients with intensive care unit-acquired pneumonia, chronic liver disease markedly increases 28-day mortality, leading to poor outcomes [14].

Among cirrhotic patients with pneumonia, *Streptococcus pneumoniae* remains the most common cause of community-acquired pneumonia (CAP) [15]. This vaccine-preventable illness causes mortality in these patients due to defects in innate immunity and pneumonia-triggered acute-on-chronic liver failure and systemic sepsis leading to multi-organ dysfunction [11]. An observational prospective cohort study determined that CAP in patients with liver cirrhosis was associated with higher mortality than in patients without cirrhosis [16]. CDC guidelines recommend that patients with chronic liver disease (CLD) aged 19–65 years who have no prior vaccination history should receive the pneumococcal vaccine [17]. Despite these recommendations, vaccination rates remain low; for instance, a study by Waghray et al reported that only 19.9% of patients had received a pneumococcal vaccination 1 year after being diagnosed with cirrhosis [18]. This highlights a critical gap in preventive care for cirrhotic patients, leaving them vulnerable to severe pneumococcal infections and associated complications.

Additionally, data on the immunogenicity of pneumococcal vaccines in patients with cirrhosis are sparse and outdated. Data evaluating outcomes of pneumococcal pneumonia in patients with cirrhosis are lacking; therefore, more studies focusing on specific parameters are needed to determine disease severity in these patients. Through this study, we aim to assess the burden and impact of pneumococcal pneumonia on various hospital outcomes among hospitalized patients with cirrhosis, using a large nationally representative database.

## Materials and Methods

### Data source

The National Inpatient Sample (NIS) database (2016–2022) was utilized to identify adult patients with cirrhosis. NIS is the largest publicly available inpatient database in the United States, managed by the Healthcare Cost and Utilization Project (HCUP) and sponsored by the Agency for Healthcare Research and Quality (AHRQ) [19]. It contains a 20% stratified sample of hospital discharges, offering reliable estimates of disease burden and outcomes. All hospitalizations in the NIS are de-identified and recorded as unique entries.

The NIS database is publicly accessible and contains de-identified patient information. The research adhered to the ethical guidelines of the responsible institution for human subject studies and complied with the principles outlined in the Declaration of Helsinki. Institutional Review Board (IRB) approval was not necessary, as the data are both publicly available and de-identified.

### Study population

Adult patients (aged > 18 years) with a diagnosis of cirrhosis were identified from the NIS database between 2016 and 2022 using International Classification of Diseases, 10th Revision (ICD-10) codes. The cohort was then divided into two distinct groups based on the presence or absence of pneumococcal pneumonia, determined by ICD-10 codes for pneumococcal pneumonia.

### Study variables

Data were collected on patient demographics (age, sex, race, primary insurance, and median income quartile) and hospital characteristics pre-specified by HCUP (region, teaching status, and bed-size). Information was also obtained on underlying liver disease etiologies such as alcohol-associated liver disease (ALD), hepatitis B, hepatitis C, metabolic-dysfunction-associated steatohepatitis (MASH), autoimmune liver disease, cholestatic liver disease, and hepatocellular carcinoma (HCC). Data regarding liver-related decompensations were also collected, including hepatic encephalopathy (HE), variceal bleeding, ascites, and hepatorenal syndrome (HRS). Data were further obtained on comorbid conditions such as renal failure, heart failure, coronary artery disease (CAD), chronic obstructive pulmonary disease (COPD), alcohol use, smoking, obesity, hypertension, hyperlipidemia, and diabetes. The modified Charlson Comorbidity Index (mCCI) was utilized to assess the comorbidity burden. This is a well-validated index based on ICD-10 CM codes used in extensive administrative data to predict mortality and hospital resource use. mCCI was calculated by removing liver comorbidities from the index [20–22].

### Study outcomes

Primary outcomes assessed were in-hospital mortality, shock, acute kidney injury (AKI), intensive care unit (ICU) admissions, and non-home discharges. Length of hospital stay and total hospitalization charges were used as surrogates for resource utilization.

### Statistical analysis

Hospital-level discharge weights from the NIS were utilized to produce national estimates. Categorical and continuous variables were compared using Chi-square tests and independent sample *t*-tests, respectively. Multivariate logistic and linear regression analysis was used to identify the impact of pneumococcal pneumonia on categorical and continuous outcomes, respectively. The regression model included patient demographics, hospital characteristics, comorbidities, disease etiology, and disease decompensation. Adjusted odds ratios (aORs) with 95% confidence intervals (CIs) were calculated. A type I error rate of less than 0.05 was deemed statistically significant. All analyses were performed using STATA version 17.0.

## Results

The analysis included 4,716,863 patients with cirrhosis. Of these, 90,680 (1.92%) were diagnosed with pneumococcal pneumonia.

### Patient and hospital characteristics

The majority of patients with cirrhosis admitted with pneumococcal pneumonia were males (62.1%), aged 45–64 years (49.9%), White (68.3%), and insured by Medicare (50.1%). A complete list of patient demographics is presented in Table 1.

**Table 1:** Patient and Hospital Characteristics Among Hospitalized Patients With Cirrhosis, Stratified by the Presence of Pneumococcal Pneumonia

Patient and hospital characteristics	Absence of pneumococcal pneumonia, n (%)	Presence of pneumococcal pneumonia, n (%)	P-value
Age category			< 0.001
18–44	536,750 (11.6)	9,235 (10.2)	
45–64	2,348,644 (50.8)	45,215 (49.9)	
> 65	1,740,789 (37.6)	36,230 (39.9)	
Sex			< 0.001
Males	2,767,419 (59.8)	56,300 (62.1)	
Females	1,858,764 (40.2)	34,380 (37.9)	
Race			< 0.001
White	3,059,494 (66.1)	61,955 (68.3)	
Black	482,105 (10.4)	9,060 (9.9)	
Hispanic	784,890 (17.0)	12,930 (14.3)	
Asian/Pacific Islander	96,610 (2.1)	1,855 (2.0)	
Native American	75,325 (1.6)	2,065 (2.3)	
Other	127,760 (2.8)	2,815 (3.1)	
Primary expected payer			< 0.001
Medicare	2,190,794 (47.4)	45,415 (50.1)	
Medicaid	1,169,564 (25.3)	23,740 (26.2)	
Private	856,705 (18.5)	14,540 (16.0)	
Uninsured	251,155 (5.4)	3,900 (4.3)	
Median household income			< 0.001
Lowest quartile	1,578,189 (34.1)	33,485 (36.9)	
Second quartile	1,231,645 (26.6)	24,895 (27.5)	
Third quartile	1,061,520 (23.0)	19,655 (21.7)	
Highest quartile	754,830 (16.3)	12,645 (13.9)	
Region of hospital			< 0.001
Northeast	791,304 (17.1)	14,015 (15.5)	
Midwest	904,525 (19.6)	20,330 (22.4)	
South	1,872,060 (40.5)	36,945 (40.7)	
West	1,058,294 (22.9)	19,390 (21.4)	
Teaching status of the hospitals			0.01
Non-teaching hospitals	1,214,614 (26.3)	24,755 (27.3)	
Teaching hospitals	3,411,569 (73.7)	65,925 (72.7)	
Bed size of hospital			< 0.001
Small	910,688 (19.7)	16,415 (18.1)	
Medium	1,329,269 (28.7)	25,655 (28.3)	
Large	2,386,226 (51.6)	48,610 (53.6)	

n (%): number of patients in each group (percentage of patients).

### Underlying liver disease and decompensations

The most common underlying liver disease among patients with pneumococcal pneumonia was ALD (42.8%) followed by hepatitis C (18.9%), and MASH (9.8%). There was a higher prevalence of HE (24.8% vs. 9.8%) and HRS (6.6% vs. 4.9%) among patients with pneumococcal pneumonia compared to those without pneumococcal pneumonia. A complete list of underlying liver disease and decompensations is provided in Table 2.

**Table 2:** Underlying Liver Disease and Liver-Related Decompensations Among Patients With Cirrhosis, Stratified by the Presence of Pneumococcal Pneumonia

	Absence of pneumococcal pneumonia, n (%)	Presence of pneumococcal pneumonia, n (%)	P-value
Underlying liver disease			
Alcohol-associated liver disease	2,112,164 (45.7)	38,800 (42.8)	< 0.001
Hepatitis C	831,415 (18.0)	17,155 (18.9)	< 0.001
Hepatitis B	93,950 (2.0)	2,065 (2.3)	0.02
Metabolic dysfunction- associated steatohepatitis	605,930 (13.1)	8,920 (9.8)	< 0.001
Autoimmune liver disease	52,645 (1.1)	815 (0.9)	0.003
Cholestatic liver disease	48,750 (1.1)	735 (0.8)	0.001
Hepatocellular carcinoma	186,835 (4.0)	2,400 (2.6)	< 0.001
Decompensations			
Hepatic encephalopathy	454,930 (9.8)	22,510 (24.8)	< 0.001
Ascites	1,923,684 (41.6)	33,925 (37.4)	< 0.001
Hepatorenal syndrome	230,100 (5.0)	5,990 (6.6)	< 0.001
Variceal bleeding	259,035 (5.6)	3,710 (4.1)	< 0.001

n (%): number of patients in each group (percentage of patients).

### Comorbidities

The prevalence of comorbidities differed in both the groups. Patients with pneumococcal pneumonia exhibited higher rates of diabetes (41.8% vs. 35.5%), renal failure (27.9% vs. 26.7%), heart failure (34.4% vs. 24.2%), and COPD (36.6% vs. 19.4%). In contrast, they had lower rates of hypertension (25.0% vs. 31.7%), hyperlipidemia (23.3% vs. 26.0%), and alcohol use (50.9% vs. 48.1%) as compared to patients without pneumococcal pneumonia. A complete list of comorbidities is presented in Table 3.

**Table 3:** Comorbidities Among Patients With Cirrhosis, Stratified by the Presence of Pneumococcal Pneumonia

Comorbidities	Absence of pneumococcal pneumonia, n (%)	Presence of pneumococcal pneumonia, n (%)	P-value
Renal failure	1,234,195 (26.7)	25,270 (27.9)	< 0.001
Heart failure	1,121,440 (24.2)	31,185 (34.4)	< 0.001
Coronary artery disease	779,060 (16.8)	15,175 (16.7)	0.71
Chronic obstructive pulmonary disease	898,120 (19.4)	33,175 (36.6)	< 0.001
Alcohol use	2,354,424 (50.9)	43,575 (48.1)	< 0.001
Obesity	785,375 (17.0)	15,275 (16.8)	0.65
Smoking	2,159,469 (46.7)	38,890 (42.9)	< 0.001
Hyperlipidemia	1,203,495 (26.0)	21,145 (23.3)	< 0.001
Hypertension	1,466,824 (31.7)	22,635 (25.0)	< 0.001
Diabetes	1,641,819 (35.5)	37,870 (41.8)	< 0.001
Modified Charlson Comorbidity Index			< 0.001
0	524,780 (11.3)	6,580 (7.3)	
1	479,650 (10.4)	10,615 (11.7)	
2	1,033,230 (22.3)	18,430 (20.3)	
3 or more	2,588,524 (56.0)	55,055 (60.7)	

n (%): number of patients in each group (percentage of patients).

### Outcomes

**Figure 1:**
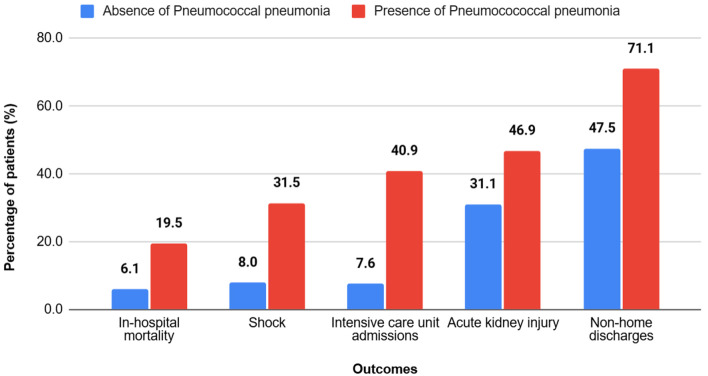
Clinical outcomes among hospitalized patients with cirrhosis, stratified by the presence of pneumococcal pneumonia. Patients with cirrhosis and pneumococcal pneumonia experienced significantly worse clinical outcomes compared with those without pneumococcal pneumonia, including higher rates of in-hospital mortality, shock, intensive care unit admission, acute kidney injury, and non-home discharge (P < 0.001 for all outcomes).

A complete list of clinical outcomes in the study population, stratified by the presence of pneumococcal pneumonia, is shown in Table 4 and Figure 1. The results of the multivariate logistic regression model for these outcomes are presented in Table 5.

**Table 4:** Clinical Outcomes and Resource Utilization Among Patients With Cirrhosis, Stratified by the Presence of Pneumococcal Pneumonia

	Absence of pneumococcal pneumonia	Presence of pneumococcal pneumonia	P-value
Outcomes, n (%)			
In-hospital mortality	280,485 (6.1)	17,655 (19.5)	< 0.001
Shock	367,780 (8.0)	28,565 (31.5)	< 0.001
Intensive care unit admissions	351,715 (7.6)	37,105 (40.9)	< 0.001
Acute kidney injury	1,438,505 (31.1)	42,530 (46.9)	< 0.001
Non-home discharges	2,195,894 (47.5)	64,435 (71.1)	< 0.001
Resource utilization, mean ± SD			
Mean length of stay (days)	6.14 ± 0.01	13.46 ± 0.12	< 0.001
Mean total hospitalization charges ($)	73,853.08 ± 503.28	195,248.1 ± 3,097.77	< 0.001

n (%): number of patients in each group (percentage of patients).

**Table 5:** Multivariate Logistic Regression Model Examining the Relationship Between Pneumococcal Pneumonia and Clinical Outcomes Among Patients With Cirrhosis

Outcomes	Adjusted odds ratio	95% Confidence interval	P-value
In-hospital mortality	2.95	2.84–3.09	< 0.001
Shock	4.75	4.58–4.93	< 0.001
Intensive care unit admissions	7.55	7.28–7.83	< 0.001
Acute kidney injury	1.85	1.79–1.91	< 0.001
Non-home discharges	2.29	2.21–2.38	< 0.001

Resource utilization	Adjusted odds coefficient	95% Confidence interval	P-value
Length of stay	6.61	6.38–6.83	< 0.001
Total hospitalization charges	112,230.50	106,802.3–117,658.7	< 0.001

#### In-hospital mortality

Patients with cirrhosis and pneumococcal pneumonia had an in-hospital mortality rate of 19.5%, compared to 6.1% in patients without pneumococcal pneumonia. The presence of pneumococcal pneumonia was independently associated with a significantly higher risk of in-hospital mortality (aOR: 2.95, 95% CI: 2.84–3.09; P < 0.001) when compared to patients without pneumococcal pneumonia.

#### Shock

The incidence of shock was markedly increased in patients with cirrhosis with pneumococcal pneumonia, compared to patients with cirrhosis alone (31.5% vs. 8.0%). On multivariate analysis, pneumococcal pneumonia was independently associated with a fourfold higher risk of developing shock during hospitalization (aOR: 4.75, 95% CI: 4.58–4.93; P < 0.001).

#### ICU admissions

The presence of pneumococcal pneumonia increased the risk of ICU admissions to 40.9% as compared to a risk of 7.6% in patients without pneumococcal pneumonia. The adjusted odds of ICU admission among patients with pneumococcal pneumonia was seven times higher in patients with cirrhosis and pneumococcal pneumonia, as compared to patients with cirrhosis alone (aOR: 7.55, 95% CI: 7.28–7.83; P < 0.001).

#### AKI

Of the patients with pneumococcal pneumonia and cirrhosis, 46.9% had AKI compared to 31.1% of patients without pneumococcal pneumonia. On multivariate analysis, after adjusting for confounding factors, patients with pneumococcal pneumonia had significantly higher odds of developing kidney injury (aOR: 1.85, 95% CI: 1.79–1.91; P < 0.001) compared to patients without pneumococcal pneumonia.

#### Non-home discharges

Around 71.1% of patients with cirrhosis and pneumococcal pneumonia were discharged to skilled nursing facilities, rehabilitation centers, or other non-home destinations as compared to 47.5% without pneumococcal pneumonia. In patients with cirrhosis, the presence of pneumococcal pneumonia was independently associated with significantly higher non-home discharges (aOR: 2.29, 95% CI: 2.21–2.38; P < 0.001) when compared to patients without pneumococcal pneumonia.

#### Length of stay

Patients with cirrhosis and pneumococcal pneumonia had a longer mean length of stay (13.46 days) when compared to patients without pneumococcal pneumonia (6.14 days). On multivariate analysis, after adjusting for confounding variables, patients with pneumococcal pneumonia had over six times increased length of stay compared to those without pneumococcal pneumonia (adjusted coefficient: 6.61 days, 95% CI: 6.38–6.83; P < 0.001).

#### Total hospitalization charges

Patients with cirrhosis and pneumococcal pneumonia had higher mean total hospitalization charges ($195,248) when compared to patients without pneumococcal pneumonia ($73,853). After adjusting for confounding variables, multivariate analysis revealed that patients with pneumococcal pneumonia had significantly higher odds of total hospitalization charges (adjusted coefficient: $112,230.50, 95% CI: $106,802.3–$117,658.7; P < 0.001) compared to patients without pneumonia.

## Discussion

Our study, using the nationally representative NIS database, gives important insights into the epidemiology, clinical burden, and outcomes of pneumococcal pneumonia among hospitalized patients with cirrhosis in the United States. Of the hospitalized patients with cirrhosis, 1.92% were diagnosed with pneumococcal pneumonia. The prevalence was lower than seen in prior studies [10, 23]. Despite regulatory bodies and hepatology societies, such as the American Association for the Study of Liver Diseases (AASLD) and European Association for the Study of the Liver (EASL), endorsing vaccination for patients with cirrhosis, vaccine uptake in routine clinical practice remains insufficient [24].

Our findings highlight that despite this being a vaccine-preventable illness, patients with cirrhosis and pneumococcal pneumonia have higher rates of mortality and adverse clinical outcomes than those without pneumococcal pneumonia infection, reinforcing the need for vaccination in this at-risk population. Cirrhosis is associated with profound immune dysfunction, predisposing patients to infections like pneumonia and conferring an increased risk of mortality once the infection occurs [23]. Defects in innate immunity in patients with cirrhosis impair pulmonary bacterial clearance and cause pro-inflammatory cytokine production [11]. Experimental studies have further shown that pulmonary clearance of pneumococci is markedly reduced in rat models with cirrhosis, likely due to reduced serum complement levels [25]. Our findings are consistent with a retrospective analysis, which found that patients with cirrhosis having pneumonia had higher mortality as compared to other infection groups [26]. On similar grounds, an infectious disease survey of 4,576 patients with cirrhosis also revealed that pneumonia was linked to a 2.95-fold increase in 30-day mortality compared to other infections [10].

In our study, ALD was the most common etiology among patients with pneumococcal pneumonia. Long-term alcohol use independently increases susceptibility to pneumococcal pneumonia and invasive pneumococcal disease [27]. In a case-control study on middle-aged adults, it was found that excessive alcohol consumption was a sole independent risk factor for CAP [28]. Compared with non-alcoholic patients, individuals with alcohol use disorder exhibited more severe clinical presentations, a higher incidence of parapneumonic effusions, and increased mortality [28]. In the current era, alcohol use still remains an independent risk factor for CAP [29–33]. Data from 1999 to 2000 from the CDC estimated that patients with alcohol use disorder are 10 times more likely to develop pneumococcal bacteremia than healthy adults [34].

Our study reports that patients with pneumococcal pneumonia have higher odds of septic shock. In a prospective cohort of hospitalized adults with CAP, patients with cirrhosis were significantly more likely to present with septic shock than patients without cirrhosis (13% vs. 6%). This demonstrates that cirrhosis is associated with a higher risk of shock in the setting of pneumonia [16]. In our study, patients admitted with cirrhosis having pneumococcal pneumonia were associated with higher odds of AKI. The development of AKI in these patients is likely multifactorial, with infection-related inflammation playing a central role. Pro-inflammatory cytokines may contribute to renal dysfunction through endothelial injury, vasoplegia, mitochondrial dysfunction, direct tubular damage, and induction of cell cycle arrest [35–41]. This is similar to a previous study in which cirrhosis patients with pneumonia had higher rates of AKI but had lower rates of renal impairment resolution and higher mortality rates of AKI as compared to other infections [42].

Our study reports that patients with pneumonia and cirrhosis have higher resource utilization than cirrhosis alone, secondary to increased ICU admissions. The length of stay, as well as the total hospitalization charges, was noted to be higher in our study. Prior research studies have demonstrated that pneumonia in patients with cirrhosis has higher rates of sepsis, organ failure, and mortality compared to other infections, reinforcing the need for early recognition and aggressive clinical management for improved patient outcomes as well as to decrease resource utilization [26, 43].

This study has some limitations which need to be acknowledged. Patients were not stratified on the basis of vaccination status and severity of cirrhosis, which might influence outcomes. Furthermore, our study lacks prognostic scoring systems to predict the mortality of cirrhosis patients with pneumonia. To account for this limitation, we used liver-related decompensations as a surrogate marker of the severity of liver disease. In addition, the use of ICD-10 coding is likely affected by testing rates, pathogen identification rates, which may lead to underdiagnosis and potential misclassification. As the NIS database only includes de-identified inpatient data, and entries represent unique hospitalizations, thus it becomes difficult to follow these patients longitudinally, including readmissions and discharge outcomes. However, the strengths include a larger cohort of patients, limiting the possibility of regional bias.

### Conclusion

Our study reports that pneumococcal pneumonia in patients with cirrhosis is associated with significantly worse clinical outcomes, prolonged hospitalizations, and increased healthcare costs. Furthermore, as concluded by prior studies, the importance of appropriate empirical antibiotic therapy in improving survival in these patients cannot be underestimated [23]. Similarly, preventive strategies such as pneumococcal vaccination may serve beneficial in reducing the incidence and infection-related morbidity in patients with cirrhosis. The impaired immunity in patients with cirrhosis increases their susceptibility to severe outcomes from pneumococcal pneumonia.

## Data Availability

The data supporting the findings of this study are publicly available and can be obtained through the HCUP website.
